# Around the Hospitals

**Published:** 1894-04-14

**Authors:** 


### Around the Hospitals.

Notice to the Managers of Institutions.—Special space is now reserved for the insertion of notices of hospital meetings and festivals. The Editor requests that all notices may reach The Hospital Office, 428, Strand, at least one week before the date of each meeting.

**The Leeds General Infirmary.**—That the new wings at the Leeds Infirmary were most necessary additions is shown by the record of patients treated. These, in the in-patients' department alone, amounted to 521 above those received in the previous year. One hundred and seventy-nine patients were also sent to various convalescent homes. In respect to funds annual subscriptions show a good increase, but contributions from the working classes show a diminution. Various societies have contributed generously, and a legacy of £1,000 has been received. The total expenditure amounted to £23,083, as against £20,054 in the previous year. All the structural improvements are not yet completed, and new machinery is needed for the laundry. Electric light has been introduced into the operating theatres. The in-patients numbered 6,299, and the out-patients 42,000.

**The Cumberland Infirmary.**—The whole report of this infirmary testifies to the efforts of those concerned in its management to render it thoroughly efficient and prosperous. The number of those ministered to has increased in both out and in-patients' departments. The Committee intend improving the children's ward, and call attention to the fact that the provision for female patients is very inadequate. A new mortuary and post-mortem room have been erected and furnished at a cost of £500. Speaking tubes, improved lighting for the wards, and new fire appliances have been added, whilst a new wing for the occupation of the nurses is almost completed. A further advance in the management of the hospital is shown by the adoption of the uniform system of accounts. The income for the year amounted to £4,551, an improvement in the average of the last ten years. Several bequests have been placed to the revenue account. The total expenditure exceeded the income by £259, the balance due to the bank amounting to £1,271. The in-patients numbered 834, the out-patients 1,844. The total cost per in-patient was slightly over £57, the cost per occupied bed being £55 8s. 10d.

**Royal Surrey County Hospital.**—That the rules of the Surrey County Hospital have been revised in respect to patients' letters in return for subscriptions needs no apology on the score of want of generosity. Subscribers of ten shillings per annum have been receiving two out-patients' letters. One is to be accorded in future, a very adequate return for a subscription so small that it would seem a pity to encourage it. The funds of the institution need augmentation, and larger contributions from all sources. A special appeal has been recently made, and other means of increasing the revenues are being adopted. We would suggest the committee to increase the interest in the institution by encouraging visitors, as few hospitals present a more pleasing aspect than the Surrey County Hospital, where floral decoration is used to great advantage, and thereby the "institutional" air yields to a homelike and cheery effect. We are glad to note in the extra expenditure on salaries that an extra nurse has been provided. A visit to the institution in previous years convinced us that it was understaffed, especially in regard to night nurses. The hospital has an unusually large number of separate wards for so small an institution, a fact that renders the nursing far more arduous, and those who have knowledge will not grudge a most necessary expenditure in the nursing department. The income for the year amounted to £4,679.

